# Association Study of rs3184504 C>T Polymorphism in Patients With Coronary Artery Disease

**Published:** 2014

**Authors:** Sarah Sadat Aghabozorg Afjeh, Sayyed Mohammad Hossein Ghaderian, Reza Mirfakhraie, Mohammad Piryaei, Hooshang Zaim Kohan

**Affiliations:** 1 Department of Medical Genetics, Shahid Beheshti University of Medical Sciences, Tehran, Iran.; 2 Department of Medical Genetics, Tehran University of Medical Sciences, Tehran, Iran.

**Keywords:** CAD, polymorphism, blood pressure, Iran

## Abstract

Cardiovascular disease has become the main factor of death and birth defects in the world and also in Iran. New clinical studies have shown that early diagnosis of patients with coronary artery disease (CAD) can contribute to effective prevention or therapeutic structures, which reduce mortality or the next chance of cardiovascular events, and increase the quality of life. Most studies on CAD disease and its genetic risk factors so far, have been done excluding the Iranian population. PubMed was used to search for all relevant studies published on or before 2013 and rs3184504 was selected for association study for CAD. A total of 200 subjects with 100 cases and 100 controls were ultimately included in the analysis. Blood samples were collected and after DNA extraction the DNA analysis was performed by TaqMan Probe Real Time PCR to evaluate the association between candidate variant with the disease and some blood biochemical factors. Our study demonstrated that there was not a direct association between rs3184504 C>T variant with risk of CAD in Iranian population, whereas, there is a significant association between this variant with increased blood LDL and diastolic blood pressure. Further molecular analysis and other disease association studies are necessary in the Iranian population.

Cardiovascular disease (CVD), including its most severe complication myocardial infarction (MI), has become the main factor of death in the world and also in Iran (1-2).

More than 80% of sudden cardiac deaths in the world are caused by atherosclerotic coronary artery disease (CAD), and the remaining 20% of cases are caused by other diseases including cardiomyopathies, congenital heart disease, left ventricular hypertrophy, aortic valve disease and other cardiac disorders. Many risk factors are involved in the familial aggregation of coronary heart disease which can be separated to environ-mental risk factors and genetic risk factors. Epidemiological studies have been done to elu Department of Medical Genetics, Shahid Beheshti University of Medical Sciences, Tehran, Iran. Sghaderian @ yahoo.co.uk cidate the risk factors of cardiovascular disease. On the other hand, other studies have focused on specific candidate genes influencing quantitative variation in lifestyle and behavioral phenotypes (3). Two separated environ-mental factors which cause the disease are common. These include smoking or other lifestyles and factors related with the location individual lives such as air pollution. Many studies investigating the association of environmental factors such as weight, body mass index (BMI), height, cholesterol level and pulse rate with blood pressure phenotypes have shown a significant association (4-5).

Macrovascular and microvascular pathology are related to cardiovascular disease such as CAD and stroke (6- 8). Studies on genetic determinants of CVD have been performed with focus on macrovascular disease traits and rare studies were conducted on the genetic analysis of microvascular disease phenotype (6, 8-9).

According to recent publications, microvasc-ular disease plays an important role in the pathogenesis of cardiovascular disease.

Noninvasive direct assessment of the human microcirculation can be done through quantitative measurement of retinal blood vessels caliber from photographs (10).

Researches demonstrate that alteration in retinal vascular caliber such as narrower arteriolar and wider venular caliber, can be related to a range of cardiovascular diseases and their risk factors (11-12), such as hypertension (13), diabetes mellitus (14-15), stroke (16), coronary heart disease (17) and cerebral small vessel disease (18-19).

An essential step in vascular remodeling and regeneration events is the migration of endothelial cells. A cascade of events which can lead to vascular disorders (e.g., atherosclerosis, vein graft arteriosclerosis and restenosis) are often initiated through the loss of endothelial function and integrity, after percutaneous revascularization and transplant arteriosclerosis (20-21).

Endothelial cell (EC) migration is a mechani-cally integrated molecular process which involves dynamic and coordinated alteration in cell attachment and cytoskeleton organization (22-23). A series of steps are required in this process including cellular extension and formation of membrane protrusion, termed lamellipodia that push the leading front and lastly, cell contraction which allows forward progression. Attachment of protrusions to the extracellular matrix (ECM) is mediated by integrins that function as receptors for cell-adhesion molecules.

As mentioned, there are also genetic risk factors causing CVD. The new strategy of genome-wide association (GWA) studies is starting to demonstrate novel genetic factors which contribute to disease risk. Many genetic variants that predispose to CVD spanning from common polymorphisms (minor allele frequency >1%) to rare, highly deleterious mutations responsible for Mendelian diseases which are usually identified by linkage studies, alone or in combination, modulate the risk of the disease. A different approach named genetic association analysis is used in order to identify the genes involved in complex diseases (24). In the case of complex diseases such as MI , linkage analysis is compounded by genetic heterogeneity of the disease and other factors such as incomplete penetrance of genes causing the disease and their interaction with environmental factors. In addition, the high prevalence of the disease-causing allele in the population, and late onset of disease can be named as another instance (25). Studies based on Meta-analysis technique published on 2011 demonstrate that specific genetic loci were introduced in association with CVD and more specifically with MI. Alteration in expression in some of them causes increasing risk of MI (25-26). Recent GWAs studies show that different

SNPs on 12q24 locus (such as rs3184504 on Lnk) are associated with platelet count, hemoglobin concentration, hematocrit and blood pressure (27). Among the studied SNPs, one case, the non-synonymous SNP rs3184504 C>T, Trp>Arg (p-value: 6E-06) in the exon 3 of SH2B3 gene had significant correlation with MI in six different populations (28). The named SNP, in addition to its companionship with eosinophil, cooperates with other increased blood parameters such as the total amount of platelets, leukocytes and red blood cells. Regarding the platelets and leukocytes the cooperation was evaluated by CAD onset (28).

Genetic diversity can be related to the regulation of blood pressure fluctuation through structural change of encoded proteins and alteration in gene expression (quantity of proteins) (29). Regarding the SH2B3 gene, based on existing studies, there is a significant correlation between the non-synonymous SNP rs3184504 and the increased diastolic blood pressure. Lnk [Src homology 2-B3 (SH2B3)] and the closely related proteins SH2B1 and SH2B2 belong to a subfamily of SH2-containing proteins with adaptor functions. These adaptor proteins are the regulators of growth factor and cytokine receptor-mediated pathways. The Lnk protein contains a pleckstrin homology (PH) domain, a NH2-terminal proline-rich region, a Src homology 2 (SH2) domain, and potential tyrosine phosphorylation sites (20, 30-31).* Lnk*−/− mice show an abnormal accumulation of erythroid cells, B lymphocytes and megakaryocytes in different hematopoietic chambers which demonstrates a defect in lymphoid and myeloid homeostasis (32-33). Protrusions attachment to extracellular matrix is led by integrin that acts as receptor for cell attachment molecules. Lnk down regulates stem cell factors and c-kit receptor signaling in B-cell precursors (32, 34-38), endothelial progenitor and mast cells (39). Deficiency of Lnk may cause increased signaling by cytokine and thrombopoietin receptors such as c-kit and c-mpl that are essential and critical for growth of hematopoietic stem and progenitor cells (20, 39). Another study performed in London confirmed association between increased LDL and rs3184504 C>T variant (40). The aim of this study was to investigate the association between CAD, increased LDL, blood pressure and rs3184504 C>T variant.

## Materials and Methods

**Patients and controls**

A total of 200 genetically unrelated Iranian subjects, comprising 100 individuals with MI and 100 normal controls from Tehran, Babol, and Mashhad cities were enrolled in the present study. All cases were selected based on the World Health Organization (WHO) criteria as described (41).

 Inclusion criteria: male and female, age over 40 years, angiography and ventriculography positive with at least one coronary artery with stenosis of 50% or more,

systolic blood pressure of ≥ 130 mm Hg, diastolic blood pressure of ≥ 85 mm Hg and/or taking medication for hypertension, triglycerides of at least 150 mg/dL (≥ 1.65 mmol/L), HDL-C less than 40 mg/dL (b 1.04 mmol/L) for men and less than 50 mg/dL (b 1.30 mmol/L) for women, fasting plasma glucose of at least 100 mg/dL (≥ 5.50 mmol/L) and waist circumference greater than 90 cm for men and greater than 80 cm for women.

Exclusion criteria: pregnant and lactating women, history of previous angiography, heart failure and history of drug use for the disease, systemic and kidney disease and history of drug use for the disease and genetic diseases influencing heart.

Normal controls were randomly selected from the same geographic area who were not suffering MI or had no family history of CAD & inflammation of the organ, with blood pressure > 140/90 mm Hg, blood cholesterol levels > 220 mg/dl, obesity, BMI ≥ 30, type 2 diabetes ,FBS > 126 mg/dl, smoking, in the past five years. Those with medical illnesses such as endocrinological abnormalities, chronic liver and/or renal diseases and those with glucose-lowering medication and alcohol consumption were excluded from the study. All participants completed a standardized questio-nnaire including information such as name, age, ethnicity, dietary habits, family history of MI, stroke, diabetes mellitus and past medical history.

**Measurements**

For all the subjects, height, weight, waist circumference, hip circumference and waist/hip ratio were measured using standardized measures and scales. The body mass index (BMI) was calculated as kg/m^2^ and a BMI of 20–25 was considered normal. Systolic blood pressure (SBP) and diastolic blood pressure (DBP) were measured using a standardized procedure. A full fasting serum lipid proﬁle including total cholesterol (TC), HDL, LDL and TG, and fasting blood glucose (FBS) concentrations were evaluated by standard enzymatic techniques. Serum C-reactive protein (CRP) concentrations were determined by polye-thylene glycol-enhanced immunoturbidimetry.

**DNA isolation and SNP selection and genotyping**

Genomic DNA was extracted from whole blood using a commercially available DNA isolation kit (High pure PCR template preparation kit, Roche company) and then was checked by agarose gel-electrophoresis for quality control and quantitated by NanoDrop ND-1000 spectrophoto-meter (Thermo Fisher Scientific, Wilmington, DE, USA). The rs3184504 of SH2B3 gene with a minor allele frequency (MAF) ≥ 0.05 were selected based on previous studies and NCBI dbSNP database (http://www.ncbi.nlm.nih.gov/). The typing of single nucleotide polymorphisms (SNPs) were determined by predesigned TaqMan SNP geno-typing assays (Real time PCR, Light cycler 96, Roche company, Germany). The reaction was performed in 25 μL ﬁnal volume with real-time polymerase chain reaction using 96-well plates on Real time PCR, Light cycler 96, (Roche Company). The polymerase chain reaction conditions were as 

follows: initial denaturation at 95 °C for 10 min then 40 cycles of denaturation at 92 °C for 15 s; and annealing and elongation at 60 °C for 1 min. Individual genotype identiﬁcation was analyzed by SDS software version 1.3 (Applied Biosystems). The genotyping success rates were greater than 95% for all SNPs. For the genotyping quality control, 10% of samples were randomly selected and measured in duplicates and the concordance rate was 100%. Nuclease-free water was used as negative control.

## Results

In the present study 100 males and females were chosen among CAD patients. Patients were diagnosed through WHO index (confirmation by coronary angiography and left ventriculography was done). Patients had shown at least one 50% or more congested vessel. In addition, 100 people aged 40 or above, who had never had heart attack or CAD in their families and risk factors such as high blood pressure, smoking, diabetes, high cholesterol level, obesity and organ inflammation with negative coronary angiography and ventriculography were chosen as control group.

Data analysis show that there was not direct significant association between C>T mutation in rs3184504 SNP with coronary artery disease (Table 1, 2)

The rate of biochemical factors such as HDL-C, LDL, triglycerides as well as systolic and diastolic blood pressure, and other factors like age, weight, sex and waist circumference had significant difference between case and control patients (Table3).

**Table 1:** Genotypes frequencies in case and control cohort

Genotype	Total	Control	Case
CC	96	44	52
CT	89	48	41
TT	15	6	9

**Table 2:** Association study between genotypes and risk of Coronary artery disease

P-Value	OR (95% CI)	Status=Control	Status=Case	Genotype	Model
0.42	1	44 (44.9%)	52 (51%)	CC	Codominant
	1.38 (0.78-2.47)	48 (49%)	41 (40.2%)	CT	
	0.79 (0.26-2.39)	6 (6.1%)	9 (8.8%)	TT	
0.39	1	44 (44.9%)	52 (51%)	CC	Dominant
	1.28 (0.73-2.23)	54 (55.1%)	50 (49%)	CT-TT	
0.47	1	92 (93.9%)	93 (91.2%)	CC-CT	Recessive
	0.67 (0.23-1.97)	6 (601%)	9 (8.8%)	TT	
0.21	1	50 (51%)	61 (59.8%)	CC-TT	Overdominant
	1.43 (0.82-2.50)	48 (49%)	41 (40.2%)	CT	
0.7	1.09 (0.70-1-70)	....	....	....	Lod-Additive

SNP association with response Status (n=200, Crude analysis)

**Table 3:** Association study of analyzed factors with coronary artery disease

Variants	P-Value
*Triglyceride*	000
HDL-C	000
LDL	015
age	000
Weight	029
Systolic Blood Pressure	000
Diastolic Blood Pressure	000
Waist Circumference	008

We also analyzed the association between rs3184504 variant with biochemical factors such as HDL-C, LDL, triglycerides, systolic and diastolic blood pressure, and other factors like age, weight, sex and some anthropometric measurements in table 4. It was shown that there were not significant associations between these factors, except for LDL and diastolic blood pressure, and non-synonymous SNP rs3184504(C>T) in case and control cohort (table 4). Regarding the SH2B3 gene and based on the present study, there was a significant association between the SNP and the increased blood LDL and diastolic blood pressure (table 4).

## Discussion

Many studies on CAD disease and its genetic risk factors have been done so far excluding the Iranian population. The locus which is proposed as risk factor for development of CAD/MI includes a candidate gene from previous studies which showed that alleles frequencies may differ in population and in genetic pool. Therefore, to determine the genetic risk factors of the disease in any population, the genetic pool of that population should be studied first. Other factors such as personal characteristics, biochemical factors and environment, can be effective on the onset and development of the disease. Some of these factors can be effective alone or in the association with other factors or with a specific mutation.

In this research only one genetic factor has been investigated. According to the results, there was not a significant correlation between the different alleles of the gene and CAD. However, a significant correlation was observed between alleles of this gene and increased LDL-C, diastolic and systolic blood pressure which will be discussed below.

One of the first studies on this SNP was done by Carr et al. in 2009 on British population through Realtime-Taqman method which demonstrated that there is not a significant correlation between alleles of C>T rs3184505 and vasculitis (the inflammation of blood vessels) which can cause inflammation in surface of blood vessels and therefore, reduction in blood circulation and even blockage of vessels (42).

In the same year, another research by Gudbjurtson et al. was performed on 12000 European and 5000 Asians which revealed that rs3184504 had association with the number of eosinophils in blood (27). Eosinophils are multifunctional leukocytes that have an important role in the beginning of inflammation responses and thus have an important role in inflammation diseases such as heart attack (28). Hematopoietic stem cells (HSCs) exist in low quantity in adult’s bone marrow. However, the question, how hemostasis of HSCs is preserved is not known clearly yet. Findings demonstrate that in the deficiency of HSCs adaptor, expression of BCL-X in comparison to normal HSCs is increased from the moment of thrombopoietin activation.

**Table 4:** Association of each polymorphic allele with different analyzed factors, using ANOVA test

		**Sum of Squares**	**df**	**Mean Square**	**F**	**Sig.**
Age	Between Groups	87.888	2	43.944	.479	.620
	Within Groups	16609.481	181	91.765		
	Total	16697.370	183			
TG	Between Groups	2321.059	2	1160.530	.279	.757
	Within Groups	751725.544	181	4153.180		
	Total	754046.603	183			
HDL-C	Between Groups	623.829	2	311.914	1.569	.211
	Within Groups	35989.988	181	198.840		
	Total	36613.817	183			
WEIGHT	Between Groups	152.324	2	76.162	.410	.664
	Within Groups	33586.589	181	185.561		
	Total	33738.913	183			
Systolic Blood Pressure	Between Groups	466.266	2	233.133	.323	.724
	Within Groups	130533.843	181	721.181		
	Total	131000.109	183			
Waist-Circumference	Between Groups	149.390	2	74.695	.581	.560
	Within Groups	23251.046	181	128.459		
	Total	23400.436	183			
Hip-Circumference	Between Groups	80.716	2	40.358	.506	.604
	Within Groups	13230.606	166	79.702		
	Total	13311.322	168			
BMI	Between Groups	52.717	2	26.358	.737	.480
	Within Groups	6469.139	181	35.741		
	Total	6521.856	183			
LDL	Between Groups	8210.898	2	4105.449	5.178	.007
	Within Groups	143495.356	181	792.792		
	Total	151706.254	183			
Diastolic Blood Pressure	Between Groups	1274.466	2	637.233	3.862	.023
	Within Groups	29868.190	181	165.018		
Total	31142.657	183			

df : Degree of freedom; F: F Statistic test; Sig: Significancy

In addition, studies show that HSCs are resistant to apoptosis because of high level of expression in BCL-XI in patients with mutation in SH2B3/LNK. As a result, the reduction of apoptosis can lead to the aggregation of HCSCs which causes several different diseases. Therefore, according to studies, SH2B3 gene is introduced as a down regulator for lymphocyte production and generally a down regulator for hemostasis. Mutation in this gene can be related to increased blood cells and blood density (43).

Signal transduction of JAK-STAT by intermediation of growth factors and cytokines has an important role in biological trends such as apoptosis, differentiation and proliferation (44). Following the attachment of cytokines to the receptors on cell surface, oligomerisation and activation of JAk (a family of tyrosine kinase) occurs (45). The family members of JAk are JAk1, 2, 3 and TYK. After activation of the cytoplasmic region of the receptor, it is phosphorylated by JAk and this action provides a region for STAT adherence. STAT is also phosphorylated and dimerised by JAK and immigrates to the cell nucleus where gene transcription is regulated. The SH2B3 gene has an important role by sticking to JAK in this process (46).

In a research done by Natali et al. in 2000, it was reported that 26% of the whole patients suffering from heart disease have had high blood pressure from which they have been suffering for approximately 9 years as well. Only 12% of them had recently been diagnosed with high blood pressure (47). In addition, two other studies on 2009 and 2010 were performed based on case control and meta-analysis methods in European and Indian populations which expressed the correlation between this SNP and high blood pressure (48). Therefore, it appears that this SNP and several other variants may contribute to the high blood pressure. The metabolism of lipids in blood such as triglyceride, increased cholesterol with low density and decreased cholesterol with high density are among the dangerous factors for coronary vessels. It is not clear whether it reveals the improvement of the coronary vessels (40).

Another study was done in London through SNP array in which the relation of this variant and the lipid blood factors such as LDL, HDL and apo B was investigated. The results demonstrated a significant correlation between this variant and increased LDL (40). As mentioned, the present study expresses the relationship and the role of this variant in diastolic blood pressure and LDL level increase.

According to several studies on CAD across the world, by the evaluation of genes from HAPMAP and GWAS data, there have been different questions raised about each of the genetic and environmental factors. One of the questions is if a genetic factor alone or in association with environment factors can lead to disease onset. This question can be answered in cases that genetic and environmental risk factors maps have been previously traced for each population. Comparison of these maps through complex and developed software can lead to individual medicine. In case of increased numbers of samples and the comparison of their results related to gene mutation, a hope for appropriate genetic map for each disease rises. Increased samples and new techniques for the diagnosis of CAD and SH2B3 gene in the future, can be helpful for such studies through complete sequencing of this gene and evaluation of methylation.

## Conflict of interest

The authors declared no conflicts of interest.
